# PERCEPTIONS OF HEALTH PROFESSIONALS, PATIENTS, AND FAMILIES ON DECISION TO DISCONTINUE LIFE-SUSTAINING TREATMENT

**DOI:** 10.1093/geroni/igad104.2925

**Published:** 2023-12-21

**Authors:** Jooseon Lee, Eunjeong Song, Dongsoon Shin, Seon Young Yun, Minjeong Eom, Suhee Oh, Heejung Lee, Rhayun Song

**Affiliations:** Chungnam National University Hospital, Daejeon, Republic of Korea; Chungnam National University Hospital, Daejeon, Republic of Korea; Chungnam National University Hospital, Daejeon, Republic of Korea; Chungnam National University Hospital, Daejeon, Republic of Korea; Chungnam National University Hospital, Daejeon, Republic of Korea; Chungnam National University Hospital, Daejeon, Republic of Korea; Chungnam National University Hospital, Daejeon, Republic of Korea; Chungnam National University Hospital, Daejeon, Republic of Korea

## Abstract

Withholding and withdrawing treatment is debated among health professionals who care for individuals at the end of life. This study aimed to compare the perception of life-sustaining treatment decisions by health professionals, patients, and families by providing data to guide medical professionals to support patients in making self-directed decisions about when to limit or continue their life-sustaining treatment. A descriptive survey was conducted with health professionals (220 from general tertiary hospitals) and 110 patients and their families. The majority of participants (85-95%) agreed with the suspension of meaningless life-sustaining treatment. In a clinical situation, the decision was made mainly by the family (84%), followed by the patient (10%) or the primary doctor (6%). However, the patients and their families reported that patients should make decisions (74%). The appropriate timing to discuss the suspension of the life-sustaining treatment was when the patients were still conscious but expecting severe deterioration” consistently reported by participants (45-58%). The most important factor during their decision-making would be “possibility of the patient’s recovery” by health professionals (43-55%), but “pain of the patients (58%)” was reported by the patients and their families. In conclusion, there is still a significant discrepancy in the perception of life-sustaining treatment decisions among health professionals, patients, and their families. The ambiguity of the timing for the decision to terminate life-sustaining treatment and the discrepancy between patients and their families’ opinions remain obstacles to implementing the Act to respect the patient’s right to self-determination and achieve the goal of a dignified end-of-life.

